# The Impact of Diet and Fibre Fractions on Plasma Adipocytokine Levels in Prediabetic Adults

**DOI:** 10.3390/nu13020487

**Published:** 2021-02-02

**Authors:** Margarita S. Dodevska, Sladjana S. Sobajic, Vesna D. Dragicevic, Ivan Stankovic, Nevena Dj. Ivanovic, Brizita I. Djordjevic

**Affiliations:** 1 Institute of Public Health of Serbia “Dr Milan Jovanovic Batut“, Center for Hygiene and Human Ecology, Dr Subotica 5, 11000 Belgrade, Serbia; 2 Department of Bromatology, Faculty of Pharmacy, University of Belgrade, Vojvode Stepe 450, 11221 Belgrade, Serbia; sladjana.sobajic@pharmacy.bg.ac.rs (S.S.S.); istank@pharmacy.bg.ac.rs (I.S.); nevenam@pharmacy.bg.ac.rs (N.D.I.); brizitadjordjevic@gmail.com (B.I.D.); 3 Maize Research Institute, Zemun Polje, S. Bajica 1, 11185 Belgrade, Serbia; vdragicevic@mrizp.rs

**Keywords:** lifestyle intervention, fibre fractions, resistant starch, obese, prediabetes, adipocytokines

## Abstract

The impact of diet and fibre fractions on adipocytokines in obese subjects with a risk of diabetes has not been investigated in detail yet. The purpose of the study is to evaluate the effects of a 12-month lifestyle intervention with different fibre profiles (resistant starch (RS)—rich fibre, or ordinary food fibre profiles) on adipocytokine levels. Fifty participants are divided into two groups (RS group and Fibre group). The groups differ only in the percentage of the recommended level of the RS consumed as a fraction of the same total fibre amount. The applied dietary intervention includes intake of 7531 KJ/daywith a total fibre portion of 25–35 g/dayfor both groups that includes 15 g/day of RS for the RS group only. The levels of leptin, adiponectin, apelin, resistin, tumor necrosis factor (TNF)-alpha and C-reactive protein (CRP) are measured, and their relationship to anthropometric and biochemical parameters is estimated. Along with significant body weight loss, only leptin is significantly reduced by 13% in the RS group while in the Fibre group, apelin levels are significant (−21%). Polynomial regression shows a negative correlation between RS intake and adiponectin (R2 = 0.145) and resistin level (R2 = 0.461) in the RS group. This study indicates the possibility that fibre fractions differently influence the outcome of lifestyle interventions, as well as their adipocytokine levels, in obese prediabetic adults.

## 1. Introduction

Obesity is a chronic disease characterised by an overly enlarged adipose tissue. Mechanisms underlying obesity are complex in nature, with evidence of the involvement of genetic and environmental factors, as well as behavioural factors [1]. There is a seven-fold greater risk of diabetes in obese individuals compared to those with healthy weights [2]. An adipose tissue is not just a simple fat deposit, it has a secretory function in producing a number of adipocytokines which themselves act as hormones affecting energy homeostasis, regulation of neuroendocrine function, and have a role in immune system response [3].

Adiponectin is an adipokine with a potential anti-atherogenic and anti-inflammatory effect and has insulin-sensitive properties [4]. Leptin is a regulator of energy homeostasis [4] and has a proinflammatory effect in diabetes and obesity [5]. Also, leptin is an activator of the immune system [6]. An adipokine with a cardio protective role is apelin [7]. Adipose tissue also releases large amounts of tumor necrosis factor (TNF)-alpha, which is at least partially responsible for developing insulin resistance in obese people [4]. Resistin is extensively secreted by monocytes and macrophages, so it may have a role as a pro-inflammatory mediator of insulin resistance [8]. The level of these adipocytokines, except adiponectin, is increased in conditions of obesity and impaired glucoregulation.

Numerous studies have confirmed that a controlled diet rich in fibre and accompanied by enhanced training has a positive effect on weight loss, which indirectly reduces the risk of chronic diseases [9,10,11,12]. Physical activity (PA) is a potent stimulus for visceral adipose tissue lipolysis and reductions in central adiposity, independent of overall weight change [13]. When applied in combination with increased PA and enriched fibre intake, dietary intervention reduces leptin [14] and TNF-alpha levels [15], while the same combination increases the adiponectin level [16]. Different fibre fractions are naturally integrated in the total fibre to demonstrate beneficial effects in the prevention of non-infectious chronic diseases (cardiovascular disease, diabetes mellitus type 2 (DMT2)), when they are consumed in pre-defined amounts [17]. Resistant starch (RS) is one of the fractions with evidence of multiple beneficial actions, such as improving insulin sensitivity [18], enhancing satiety and normalisation of blood cholesterol [19]. The impact of diet and fibre fractions on adipocytokines in obese subjects with a risk for DMT2 is not researched in detail. We hypothesise that dietary intervention with different fibre fractions will affect anthropometric parameters and, thus, adipocytokine levels. Therefore, the objective of this study is to observe how a long-term dietary intervention with reduced calories, increased fibre intake, and enhanced PA affects the anthropometric parameters in obese individuals with impaired glucoregulation, and its subsequent effect on leptin, adiponectin, resistin, apelin, and inflammatory markers. Additionally, the study also investigates the differential effects of two RS levels, as well as the intake of other fibre factions (arabinoxylan, fructan, beta-glucan, and cellulose), as a part of a dietary and lifestyle intervention on leptin, adiponectin, resistin, apelin, and inflammatory markers.

## 2. Materials and Methods

### 2.1. Participants

**Figure 1:**
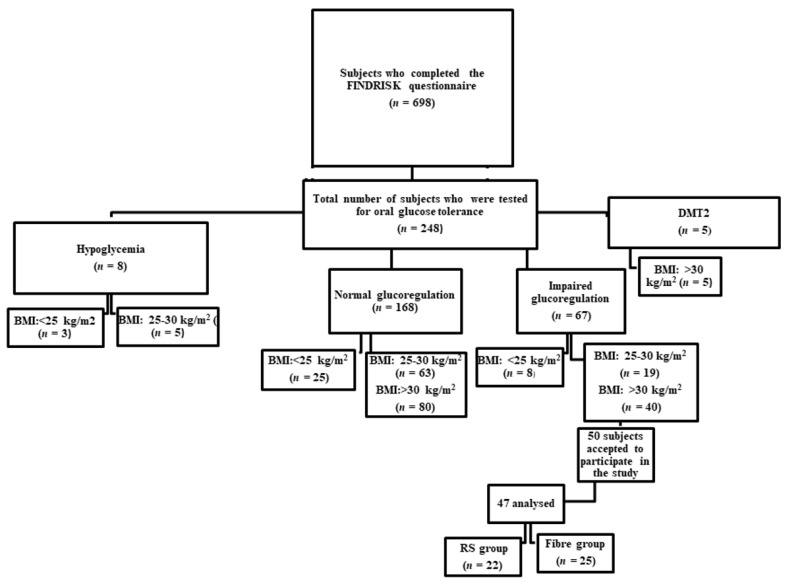
Flow diagram of participants recruiting.

The participants in the study were recruited based on the results obtained by a FINDRISK questionnaire [9]. A group of 698 subjects completed the FINDRISK questionnaire and 248 subjects who obtained ≥ 11 points were further tested for oral glucose tolerance (OGT). Subjects with impaired glucoregulation and who were overweight/obese were offered an opportunity to participate in the study (Figure 1).

### 2.2. Design

The subjects included in this study were not dependent on diabetic drugs. Fifty patients aged 45–74 enrolled in the study and were randomly assigned to the resistant starch (RS) group and the Fibre group. The subjects were contacted at least twice a month and regular control visits to a physician were conducted every 3 months. Lifestyle intervention lasted 12 months. The study protocol has been published previously [19] and was conducted according to the guidelines laid out in the Declaration of Helsinki. All procedures involving human subjects were approved by the Ethical Committee for Clinical Investigations at the Faculty of Pharmacy, Belgrade University (protocol code: 1585/2, date of approval: 17 September 2012). Written informed consent was obtained from all subjects. The study was registered with ANZCTR.org.au., identifier no. ACTRN12613001118796. Socio-demographic characteristics of the study population are presented in Table 1.

### 2.3. Dietary Recommendation

During the investigated period, calorie and macronutrient intakes were altered in the groups according to a current recommendation aimed at lowering diabetes risk factors [20]. Recommendations included a regimen of 7531 KJ/day (1800 Kcal day^−1^), increased PA to ≥30 min/day, reduced fat intake to <30% of total energy, saturated fat at <10% of energy, and to increase fibre intake to 25–35 g/day for the participants. The single difference between the two study groups was the amount of RS in their diets. The RS group was advised to include higher quantities of plant-based food rich in RS, especially legumes and cereals [21,22,23], to provide approximately 15 g/day of RS, while the Fibre group did not have any specific recommendations related to the intake of RS-rich food but received general advice to consume plants rich in fibre. Table 2 shows the food spectrum (without legumes and cooked cereals), recommended to subjects from both groups participating in the lifestyle intervention. No additional fibre or other supplements were used during the study.

### 2.4. Physical Activity

The participants in this study completed the validated Kuopio Ischemic Heart Disease Risk Factor Study 1 year Leisure Time Physical Activity (LTPA) questionnaire at the beginning and the end of the study [24]. Low PA included a minimum of 4 h a week (e.g., walking, cycling, light gardening, fishing, hunting), but excluded travel to work. Moderate PA included exercises like running, swimming, other sports, and heavy gardening, for a minimum of 3 h a week. The duration (minutes per week) of low and moderate-intensity LTPA was calculated. All participants were instructed to practice 30 min of daily exercise.

### 2.5. Food Composition Analysis

The nutrients were analysed for the percentage of moisture, proteins, fats, dietary fibre, and ash. The nutritional value was determined using the procedures described by the Association of Official Analytical Chemists [25]. Dietary fibre was determined using a gravimetric method [26]. Total carbohydrates were calculated as the residual difference after subtracting protein, ash, moisture, total fibre, and crude fat content.

Total energy was determined by the calculation of energy values of carbohydrate, fat, protein, and fibre: Energy, KJ = 37 (crude fat content, g) + 17 (protein content, g + carbohydrate content, g) + 8 (fibre content, g). Cellulose determination was performed according to SRPS ISO 6541:1997 [27]. Resistant starch, beta-glucan, arabinoxylan, fructan, and sugars were quantified by a spectrophotometric method using enzymatic assay kits, Megazyme, Bray, Ireland. Analyses were performed according to an instruction manual of a kit producer [28,29].

Fibre fractions intake were calculated using 3-day food records, applying data on the fructan, arabinoxylan, cellulose, resistant starch, and beta-glucan content in the foods from Table 2 which were previously analysed for fibre and RS content [22,23,30].

### 2.6. Anthropometric and Biochemical Parameters

Height, weight (Body Mass Index (BMI) calculated), waist circumference, and blood pressure were measured by standard procedures. Blood samples were taken at the Clinic for Endocrinology, Diabetes and Metabolism Disorders of the Clinical Centre of Serbia. Blood biochemistry tests and routine laboratory analyses of blood samples were performed at a clinic laboratory in duplicate. The values of C-reactive protein (CRP) were analysed by a turbidimetric immunoassay method on an Olympus AU 400 (Olympus, Hamburg, Germany). The intra-assay coefficient of variation (CV) was 1.5%, and the inter-assay CV was 2.3%.

### 2.7. Analysis of Adipokines

Plasma leptin was measured using a sandwich enzyme-linked immunosorbent assay (ELISA) (DRG diagnostics, Marburg, Germany). Intra- and inter-assay coefficients of variation (CVs) were <6% and <9%, respectively, for the sample concentration range of 1–100 ng/mL. The detection limit was 1.0 ng/mL. Plasma adiponectin was measured using the ELISA technique (MBL International Corporation, Massachusetts, MA, USA). Intra- and inter-assay CVs were <4.4% and <4.3%, respectively. The detection limit was 1.455 ng/mL. Plasma resistin was measured using the ELISA technique (Alpco Diagnostics, Salem, USA). Intra- and inter-assay CVs were <5.2% and <7.0%, respectively. The limit of detection was 100 pg/mL. Plasma tumor necrosis factor (TNF)-alpha was measured using the ELISA technique (BioLegend, San Diego, USA). Intra- and inter-assay CVs were <6% and <8%, respectively. The detection limit was 4 pg/mL.

### 2.8. Statistical Power

The minimum number of participants required for the study was twenty-five, with the aim to decrease the blood fasting glucose after dietary intervention by approximately 10% (0.064 mmol/L and 0.8 standard deviation (SD)), with alpha (α) = 0.05 and 1–beta (β) = 0.8 [31].

### 2.9. Statistical Analysis

To compare baseline characteristics between the assigned groups, SPSS version 20.0 (Chicago, IL, USA) was used. Means and standard deviation (SD) were calculated for continuous variables with *p* values using independent *t*-tests for the between-group comparison, while the within-group comparison between the baseline and after 12 months was performed using paired *t*-tests. A Shapiro-Wilk test was used for normality testing. Concerning results that did not fit, normal distribution, the median, and inter-quartile range were reported with *p* values calculated using the Wilcoxon’s rank-sum test for the between-group comparison, and the Wilcoxon’s signed-rank sum test for the within-group comparison. The dependency of categorical variables was analysed using a Chi-squared test. The dependence between fibre, i.e., RS with leptin, adiponectin, resistin, and apelin, was presented as a polynomial regression. A principal component analysis (PCA) was used for the evaluation of interdependence between food components (total fibre and fibre fractions) and biochemical parameters (leptin, adiponectin, resistin, apelin, TNF-alpha and CRP), as well as between anthropometric parameters (body mass index (BMI), body weight, and waist circumference) and adipokine levels. All results were statistically significant with *p* values < 0.05.

## 3. Results

### 3.1. Participant Recruiting and Dietary Regimen Design

**Figure 2:**
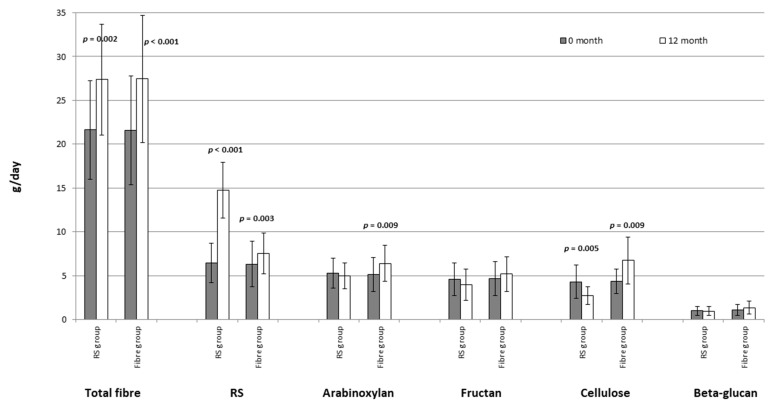
Fibre and fibre fractions intake at the beginning and after 12 months of lifestyle intervention in the resistant starch (RS) and the Fibre group; (Means ± SD; paired *t*-tests).

Fifty obese participants aged between 45–74 with impaired glucoregulation were enrolled in the study and were monitored throughout a one year study duration. The subjects were divided into two groups (25 individuals in the RS group and 25 individuals in the Fibre group). Lifestyle intervention included dietary changes and enhanced exercise. Three individuals from the RS group prematurely terminated the recommended dietary regimen complaining of the lack of time necessary to prepare a meal. A total of 47 obese subjects finished the study. During the 12 months of the study, total fibre intake increased by 21% in both groups, but RS intake in the RS group was 2.2-folds higher than in the Fibre group (Figure 2). Intake of arabinoxylan was significantly increased only in the Fibre group (*p* = 0.009), while intake of cellulose was increased in the Fibre group, (*p* = 0.009), but decreased in the RS group, (*p* = 0.005), (Figure 2).

### 3.2. Changes in the Anthropometric Parameters

**Figure 3:**
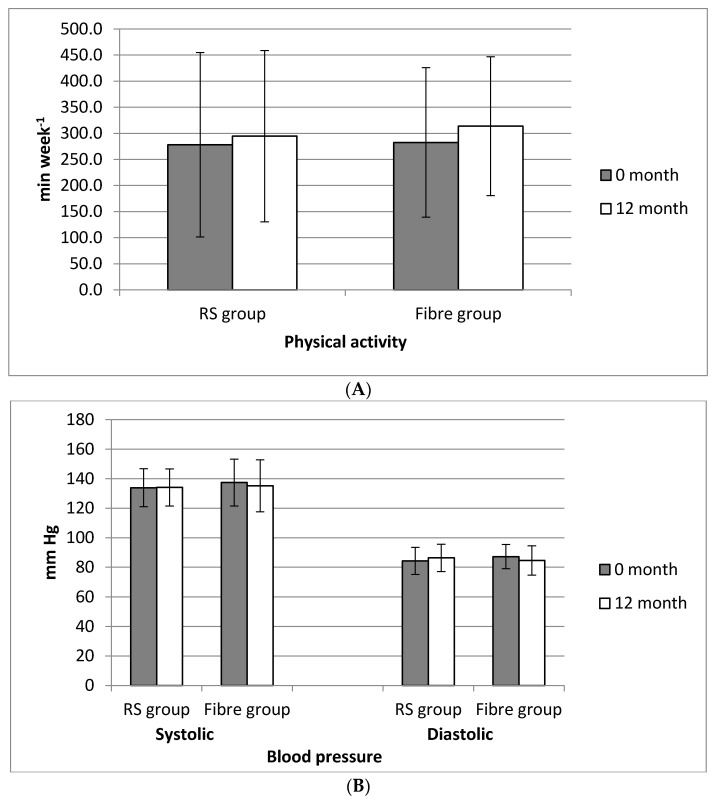
(**A**) Physical activity and (**B**) blood pressure in the subject groups (RS group and Fibre group), at the beginning and after 12 months of lifestyle intervention.

After a 12-month period of lifestyle intervention, several dietary, anthropometric, and biochemical factors were statistically changed: body weight, and calories decreased slightly more in the RS group (4.6% (*p* ≤ 0.001); 6.1% (*p* = 0.019), respectively), than in the Fibre group (2.7% (*p* = 0.001); 5.3% (*p* = 0.024), respectively). The same decreasing trend was noticed for the Body Mass Index (BMI) and waist circumference: 4.2% (*p* = 0.005); 3.5% (*p* = 0.003), respectively, in the RS group, and in the Fibre group 2.0% (*p* = 0.002); 3.0% (*p* ≤ 0.001), respectively). The fasting plasma glucose showed no significant changes in both groups at the end of the study, while the glucose level after a two-hour oral glucose tolerance (OGT) test was decreased by 12% only in the Fibre group (*p* = 0.034). There was no significant difference between the groups in systolic and diastolic blood pressure at the beginning and the end of the study (Figure 3A). Physical activity (PA) was calculated and in the RS group it was 278 min/week and in the Fibre group 283 min/week at the beginning of the study while, at the end of the study, the total PA level increased by 5.9% in the RS group (*p* = 0.640) and 11.0% in the Fibre group (*p* = 0.265); the final PA level again was not significantly different between groups (*p* = 0.659), Figure 3B.

### 3.3. The Interdependence between Fibre and Its Fractions, Adipokine Levels, and Pro-Inflammatory Markers

**Figure 4:**
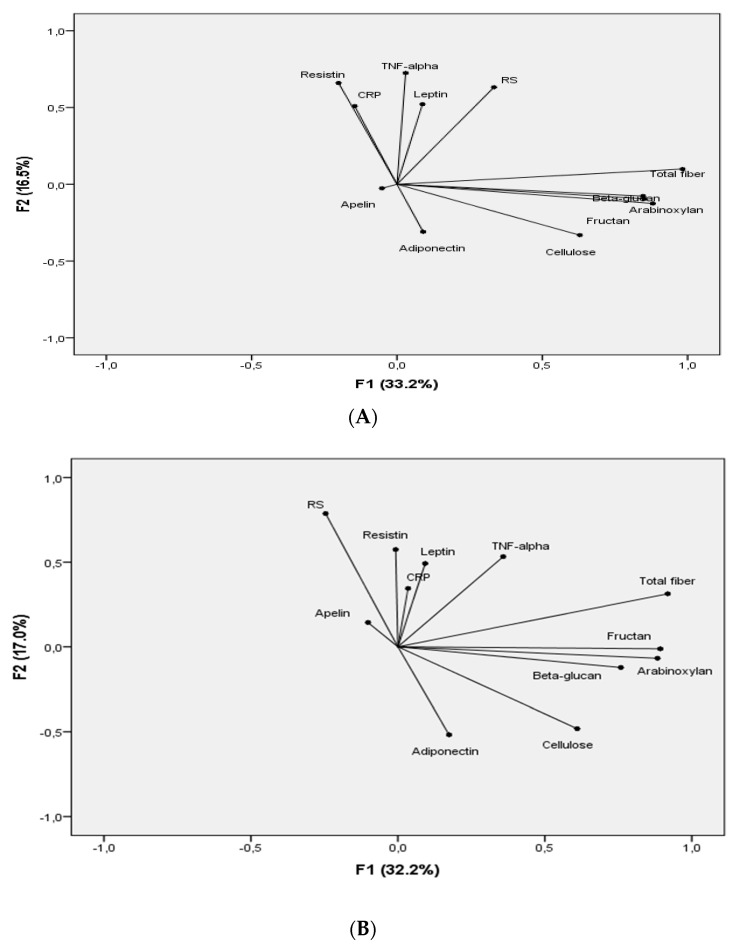
Principal Component Analysis for total fibre, fibre fractions (fructan, β-glucan, arabinoxylan, cellulose and resistant starch (RS) and adipocytokines content at the beginning (**A**) and after 12 months (**B**) of lifestyle intervention.

A PCA was used to integrate the results of dietary intervention present through increased RS, i.e., fibre intake and biochemical parameters, to discover the possible correlations among measured parameters and to classify the parameters in a factor plane. Occurring at the beginning of the experiment, PCA revealed that two axes participated in total variability with 49.7% (factor–axes (F)1: 33.2% and F2: 16.5%) and, after 12 months, the axes participated in total variability with 49.2% (F1: 32.2% and F2: 17.0%). According to the PCA, total fibre, fructan, β-glucan, arabinoxylan, and cellulose correlated mainly with the first axis—factor (0.982, 0.880, 0.851, 0.846, and 0.629, respectively), while TNF-alpha, resistin, RS, leptin, and CRP were mainly connected to the second axis—factor (0.725, 0.660, 0.632, 0.522, and 0.509, respectively), Figure 4A. After 12 months of the study, F1 was again determined using total fibre, fructan, arabinoxylan, β-glucan and cellulose (0.918, 0.893, 0.883, 0.759, and 0.610, respectively), whereas F2 was determined using RS, resistin, TNF-alpha, and adiponectin (0.788, 0.575, 0.533, and −0.518, respectively), Figure 4B. Only adiponectin correlated negatively.

**Figure 5:**
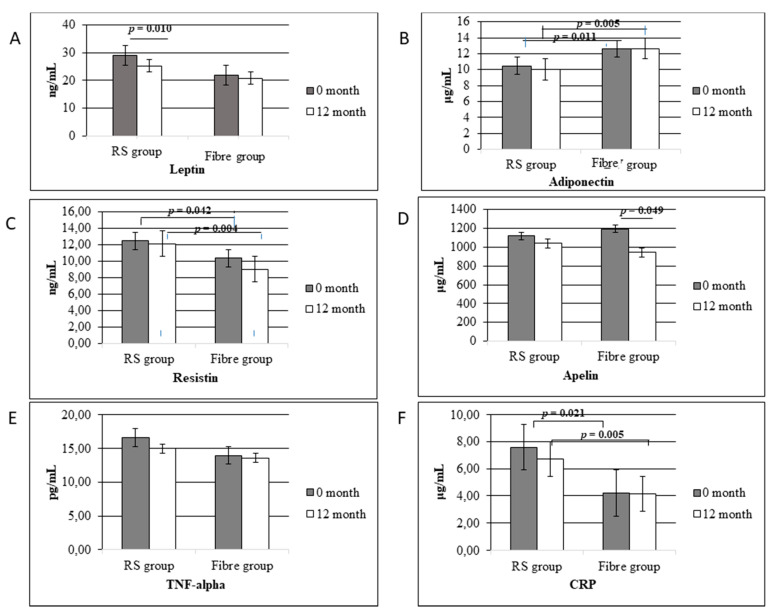
Plasma levels of adipokines: (**A**): leptin, (**B**): adiponectin, (**C**): resistin, (**D**): apelin) and factors of inflammation: ((**E**): TNF-alpha, (**F**)): CRP in the subject groups at the beginning and after dietary and lifestyle intervention.

The results of adipokine measurements for both groups at the study initiation and after 12 months are presented in Figure 5. Starting leptin levels in the groups did not differ but, after 12 months, a significant decrease of 13% in the RS group was noticed (*p* = 0.010), which was not the case in the Fibre group. Dietary intervention in the Fibre group decreased resistin (13%) and significantly decreased apelin (20%, *p* = 0.049) levels, which was not noticed in the RS group. The values for adiponectin and resistin were significantly different between groups (RS group and Fibre group) at the baseline (*p* = 0.011 and 0.042, respectively), as well as at the end of the intervention (*p* = 0.005 and 0.004, respectively). However, there was no statistically important difference in the adiponectin and resistin levels within the same groups between the baseline and at the end of the study. Besides adipokines, two inflammatory factors also were analysed. Before the lifestyle intervention, TNF-alpha levels between groups were not statistically significant (*p* = 0.208), while values for CRP significantly differed (*p* = 0.021). After 12 months of intervention, TNF-alpha decreased by 6% and 14% in the RS and the Fibre group, respectively, while CRP values decreased by 32% only in the Fibre group.

A linear regression model (Table 3) was used to test whether adipokine levels were related to anthropometric parameters after one year of intervention. A starting linear regression model revealed significant correlations only between the Body Mass Index (BMI) and leptin plasma levels in both groups (RS group, β = 0.632 [95% CI 0.111, 0.456; *p* = 0.003]; Fibre group, β = 0.677 [95% CI 0.121, 0.397; *p* = 0.001], (values not shown in Table 3). After 12 months, plasma leptin still correlated to BMI in the RS and the Fibre group (β 0.595 [95% CI 0.227, 1.417]; *p* = 0.010) and (β 0.799 [95% CI 0.852, 1.629]; *p* < 0.001), respectively). Furthermore, a significant correlation between body weight and leptin was confirmed in the RS group (β 0.458 [95% CI 0.031, 1.099]; *p* = 0.039).

Data are β-coefficients (95% CI) from the linear regression models for associations between cytokine levels after 12 months of dietary interventions and anthropometric parameters (body mass index, body weight, and waist circumference).

The results of lifestyle intervention, i.e., interdependence between anthropometric parameters and adipokine levels at the beginning and end of the study, were analysed using PCA. F1 contributed with 59.2% and F2 with 32.5% in total variability (Figure S1). Leptin, adiponectin, fasting glucose and BMI correlated significantly and positively, while apelin correlated negatively with F1. Also, body weight and waist circumference correlated positively with F2. After 12 months of experimentation, fasting glucose varied mostly in the RS group, while apelin, body weight and waist circumference varied mostly in the Fibre group. Polynomial regression presented a total fibre intake ≥30 g/day in the RS group was in negative correlation with resistin (R^2^ = 0.185). Fibre intake had no significant influence on adiponectin, leptin, and apelin, while RS intake ≥ 15 g/day was negatively correlated with resistin (R^2^ = 0.461), and adiponectin (R^2^ = 0.145, Figure S2A,C). Concerning the Fibre group, fibre intake and RS intake had no significant effect on all adipokines (Figure S2B,D).

## 4. Discussion

### 4.1. The Impact of Lifestyle Changes on the Anthropometric Parameters

The healthful effect of a 12-month intervention with reduced calorie and increased fibre intake, with enhanced Physical Activity (PA) in obese individuals with impaired glucoregulation was evaluated in detail in our previous report [19]. The impact of a recommended diet enriched with total fibre in both groups, and an increased intake of RS in the RS group, followed by a small increase in PA, have resulted in reduced body weight, Body Mass Index (BMI), and waist circumference. Reduced calorie intake along with enhanced PA was shown by other authors to be beneficial for the general health status in obese individuals with diabetes mellitus type 2 (DMT2) [20]. The American Diabetes Association has given these recommendations related to lifestyle modification, among which a controlled dietary regimen through lowering fat and calorie intake and enhancing exercise (walking briskly for at least 30 min/day, for a minimum of five days weekly) are particularly emphasised. During our study, approximately 2092 KJ (500 kcal)/day reductions from the usual calorie intakes were recommended for both groups. This goal was not achieved after a year, but the calorie intake was significantly lower. Regular exercise also has an important role in obesity and DMT2. Exercise alone, diet alone and particularly, their combination was equally effective in reducing the progression from impaired glucoregulation to DMT2 [32]. After 12 months, a PA increase was noticeable in both our study groups, but without statistical significance.

The authors believe that one important additional reason for this study showing a small but important effect on body weight, BMI, and waist circumference in both groups of subjects who were enrolled in it was the direct and frequent communication between researchers and all subjects throughout the study period resulting in a high level of motivation and compliance. This kind of counseling also was effective for subjects from the intervention group of the Finnish Diabetes Study [10] who had seven sessions with study professionals within the first study year, where the lifestyle and dietary intervention also was based on given recommendations.

### 4.2. Adipokine Alterations Caused by Lifestyle Intervention

Considering that applied lifestyle intervention resulted in a statistically significant decrease in body weight, Body Mass Index (BMI), and waist circumference in obese participants, our further investigation was focused on adipokine level changes.

#### 4.2.1. Leptin

The first adipokine of interest in obese patients was leptin. Body weight reduction is accompanied by a decrease in plasma leptin [4]. During the present study, the combined effects of diet and exercise reduced waist circumference as well as body weight in both groups of participants, although the more pronounced effect was in the RS group. Other investigations also support that lifestyle changes could decrease the leptin level [4,33]. During this research, a decrease in the leptin level was noticed, significantly in the RS group, but was insignificant in the Fibre group. Observed changes in leptin concentration mirrored changes in body weight, particularly in the RS group where leptin levels correlated positively with body weight and BMI whereas, in the Fibre group, it correlated positively only with BMI. The influence of fibre on leptin levels was investigated in young Japanese women where an increased fibre intake was associated with lower plasma leptin values, independent of potential confounding factors, including BMI [33]. There are no available studies that report the impact of particular fibre fractions on leptin blood measurements. Considering the fact that RS improves the feeling of satiety [18], which leads to lower food intake, while leptin declines along with a reduction in calorie intake, we hypothesis that RS is more efficient by acting indirectly on the reduction of body weight.

#### 4.2.2. Adiponectin

Epidemiological studies conducted on obese individuals provide evidence for the beneficial effect on adiponectin levels of food high in fibre, such as fruits, wholegrain cereals, cereal fibre, as well as moderate alcohol consumption, often referred to as a Mediterranean diet, or from another healthy dietary pattern [34,35]. After one year of both interventions, adiponectin levels mainly remained unchanged, although a significant increase in total fibre intake was achieved by both groups. A possible explanation may be found in the fact that subjects from both groups consumed low levels of whole grain food in their diets, whereas the most positive effect on adiponectin levels was achieved with whole grain fibre [36] and could be supported to some extent by a weak interdependence between adiponectin and cellulose, found via PCA. This confirms the results from a polynomial regression where increased RS intake was negatively correlated with adiponectin. Several studies indicated that diets with only reduced caloric intake that resulted in decreased body weight had almost no effect on the adiponectin levels [37,38], except when body weight loss was >7% [39]. This means that an expected change in adiponectin levels in our study could be addressed by a moderate body weight loss. It was shown that minor changes in body weight led to a less reduction in body fat, adipose tissue, and the size of adipocytes, which is probably the most relevant location of adiponectin production and secretion [40].

#### 4.2.3. Apelin

Apelin is another adipokine of interest and it was decreased in both groups, but statistical significance was noticed only in the Fibre group. The association between apelin levels, glucose concentrations, and insulin sensitivity provided evidence that apelin may have a role in the pathogenesis of diabetes mellitus type 2 (DMT2) [41]. Concerning the RS group, the body weight reduction was more pronounced but, in the Fibre group, a statistically significant decrease in glucose after a two-hour oral glucose tolerance (OGT) test was noticed, which was described in detail in the previous paper [19]. The value of apelin indicated that apelin had a more significant role in an improvement in glucoregulation than in body weight reduction. Total fibre intake had a not statistically significant correlation with apelin levels in both groups.

#### 4.2.4. Resistin

Identical to the apelin findings, resistin levels are also increased in overweight/obese subjects with impaired glucoregulation [8]. Azuma et al. [42] found a proportional decline in resistin level concomitant to the reduced extent of fat mass and visceral fat tissue, resulting in changes in glycemia and insulin secretion. The effect of fibre on resistin levels is still not investigated. One of the rare studies that examined changes in resistin, adiponectin, and leptin in relation to increased arabinoxylan intake demonstrated no noticeable effect [43]. The results of polynomial regression in the RS group show a negative correlation between the resistin level and RS intake ≥ 15 g/day. Other than that, the resistin level decreased in the Fibre group, but not significantly. While in the RS group the reduction of body weight was more evident, in the Fibre group the process of glucoregulation was more pronounced, so it could be supposed that decreased oral glucose tolerance (OGT) glucose levels, which were seen only in the Fibre group, were more important for lowering resistin levels after 12 months, as it was seen with apelin.

### 4.3. The Impact of Lifestyle Intervention on Pro-Inflammatory Markers

Besides adipokines, pro-inflammatory markers TNF-alpha and CRP have been shown to be disturbed in chronic diseases such as obesity and impaired glucoregulation. A large waist circumference also is associated with increased TNF-alpha circulation levels. Gacka et al., [44] found higher concentrations of TNF-alpha in patients with diabetes mellitus type 2 (DMT2) in comparison to patients with normal glucoregulation. Similarly, Gokulakrishnan et al., [45] concluded that TNF-alpha concentration correlates to Body Mass Index (BMI) and impaired glucose metabolism and, even more, they found that pro-inflammatory markers, CRP and TNF-alpha, demonstrate a cumulative effect on atherosclerotic changes in patients with obesity and normal glucoregulation. It is well known that body weight reduction increases insulin sensitivity, and it is considered that decreased secretion of adipose TNF-alpha is partly involved in this mechanism [4,46].

Weight loss, rather than only increased Physical Activity (PA), is the lifestyle factor that is primarily responsible for improvement in the inflammatory profile [15]. During our study, inflammation decreased concomitantly with lowering obesity, i.e., central obesity in particular, however insignificantly. Achieved changes in waist circumference and body weight in both groups were moderate and insufficient to significantly reduce inflammatory biomarkers. Fibre type had no significant effect on achieved weight loss, so it was not sufficient to induce a decrease in inflammatory markers. Studies investigating the effect of fibre on inflammatory markers are rare. Bodingham et al., [47] conducted a study where a statistically significant TNF-alpha decrease was demonstrated in obese individuals with RS supplementation, which was given in a quantity that reached almost 40 g/day.

The present study has several limitations. Primarily, the enrolled subject number was insufficient for more decisive conclusions. Secondly, groups slightly differed in several parameters at the beginning of the study. Last but not least, the control of the study conditions and the compliance of the participants could have been conducted in a more regular fashion.

The strength of the study was a low drop-out level despite its long-term duration. This study confirms that long-term lifestyle intervention, although with moderate effect on anthropometric and biochemical parameters, influences adipocytokine in obese individuals with impaired glucoregulation. The obtained results also indicate the possibility that fibre fractions differently influence the outcome of lifestyle intervention, requiring further investigation with more controlled conditions.

## 5. Conclusions

To conclude, a lifestyle intervention comprising diet with increased total fibre intake with a low RS content, has clearly demonstrated a beneficial effect on anti-inflammatory (apelin) levels, while increased RS within the total fibre intake led to a decrease in leptin levels in obese patients with impaired glucoregulation. The influence of total fibre on the apelin level in the RS group was probably absent due to a reduced amount of other fibre fractions regarding a high RS level from natural sources. Also, increased RS intake via the application of regular food combinations was used, as far as we know, for the first time in fibre intervention studies.

## Figures and Tables

**Table 1:** Socio-economic and lifestyle characteristics of the sample population at the beginning of the study.

Characteristics	RS Group (*n* = 25)	Fibre Group (*n* = 25)	*p*
Men	8	9	0.765
Education Level			
Primary	8	11	
Secondary	6	8	
College and University	9	6	0.585
Smoking Status			
Current	12	15	0.395
Alcohol Consumption			
Zero	14	17	
Low	11	8	0.382
Physical Activity			
Low	15	12	
Moderate	10	13	0.725

A comparison of qualitative variables between groups was performed using the Chi^2^ test.

**Table 2:** Recommended fibre/resistant starch-rich foods in the dietary intervention.

Nutrient	Energy kcal/100 g	Fat g/100 g	Protein g/100 g	Carbohydrate g /100 g	Sugar g/100 g	Total Starch g/100 g	Total Fibre g/100 g	Cellulose g/100 g	Beta-Glucan g/100 g	Arabinoxylan g/100 g	Resistant Starch g/100 g	Fructan, g/100 g
Foods High in Resistant Starch												
Rye bread	259.0 ± 18.9	2.5 ± 0.3	7.8 ± 0.6	48.9 ± 2.4	2.0 ± 0.3	48.6 ± 0.9	5.0 ± 0.3	0.4 ± 0.2	0.3 ± 0.1	1.3 ± 0.2	2.0 ± 0.4	1.4 ± 0.2
Corn bread	253.6 ± 13.6	1.8 ± 0.3	6.1 ± 0.6	51.6 ± 2.0	1.5 ± 0.2	50.1 ± 2.7	3.3 ± 0.4	0.2 ± 0.1	0.2 ± 0.2	0.8 ± 0.2	1.2 ± 0.2	0.9 ± 0.3
Wholemeal bread	238.4 ± 9.1	1.4 ± 0.4	8.9 ± 0.6	45.5 ± 2.1	0.9 ± 0.2	45.0 ± 2.8	4.5 ± 0.5	0.7 ± 0.2	0.2 ± 0.1	1.1 ± 0.3	1.1 ± 0.2	1.0 ± 0.2
Potato salad	115.9 ± 6.7	4.8 ± 0.4	2.5 ± 0.1	14.0 ± 0.5	0.5 ± 0.2	13.2 ± 0.4	3.6 ± 0.4	0.1 ± 0.1	n.d.	n.d.	3.5 ± 0.4	n.d.
Rice pudding	111.8 ± 9.8	1.6 ± 0.1	3.5 ± 0.6	20.6 ± 1.4	4.0 ± 0.4	16.5 ± 0.9	0.7 ± 0.3	n.d.	n.d.	0.1 ± 0.1	0.6 ± 0.2	n.d.
Pasta	126.5 ± 7.6	1.3 ± 0.3	4.1 ± 0.4	23.1 ± 0.6	0.9 ± 0.2	22.2 ± 0.6	3.1 ± 0.4	0.2 ± 0.1	n.d.	0.6 ± 0.1	1.6 ± 0.3	0.5 ± 0.1
Polenta	112.5 ± 8.0	1.1 ± 0.2	2.3 ± 0.3	22.0 ± 1.1	0.9 ± 0.1	21.1 ± 0.6	2.8 ± 0.3	0.3 ± 0.1	n.d.	0.2 ± 0.1	1.7 ± 0.2	0.4 ± 0.1
Rye flakes with yogurt	163.8 ± 10.2	1.5 ± 0.2	6.5 ± 0.6	27.7 ± 1.2	4.0 ± 0.1	23.6 ± 1.9	7.0 ± 0.8	0.6 ± 0.1	0.7 ± 0.2	2.8 ± 0.3	1.6 ± 0.3	1.8 ± 0.2
Barley flakes with yogurt	177.8 ± 14.5	1.6 ± 0.2	6.7 ± 0.4	31.7 ± 2.3	5.9 ± 0.4	25.8 ± 1.4	5.1 ± 0.6	0.2 ± 0.1	1.8 ± 0.3	1.3 ± 0.3	1.0 ± 0.2	0.6 ± 0.1
Oat flakes with yogurt	186.3 ± 8.8	3.7 ± 0.1	6.5 ± 0.4	28.8 ± 1.2	4.3 ± 0.3	24.6 ± 1.2	6.0 ± 0.5	0.5 ± 0.2	1.9 ± 0.4	1.1 ± 0.2	0.2 ± 0.1	0.2 ± 0.1
Banana (green)	87.7 ± 8.3	0.5 ± 0.2	1.1 ± 0.2	17.0 ± 1.0	9.2 ± 0.9	7.9 ± 1.1	5.5 ± 0.9	0.6 ± 0.3	n.d.	0.2 ± 0.1	4.3 ± 0.6	0.1 ± 0.1
Foods High in Fibre												
Baked pumpkin	24.1 ± 2.8	0.1 ± 0.1	0.9 ± 0.2	4.3 ± 0.3	2.8 ± 0.6	1.5 ± 0.4	1.2 ± 0.3	0.4 ± 0.1	n.d.	n.d.	0.5 ± 0.2	0.1 ± 0.1
Carrots	32.7 ± 3.3	0.2 ± 0.1	0.8 ± 0.1	5.4 ± 0.4	3.9 ± 0.7	1.5 ± 0.3	3.1 ± 0.4	1.6 ± 0.3	n.d.	0.1 ± 0.1	0.5 ± 0.2	0.3 ± 0.1
Chard salad	23.9 ± 3.6	0.9 ± 0.1	1.9 ± 0.3	1.1 ± 0.3	1.1 ± 0.1	n.d.	2.1 ± 0.3	1.0 ± 0.3	n.d.	0.1 ± 0.1	n.d.	0.1 ± 0.1
Cabbage salad	33.7 ± 6.0	1.2 ± 0.3	1.4 ± 0.3	3.0 ± 0.4	3.0 ± 0.4	n.d.	2.5 ± 0.4	1.2 ± 0.2	n.d.	0.1 ± 0.1	n.d.	0.1 ± 0.1
Broccoli salad	44.8 ± 5.4	1.2 ± 0.2	2.9 ± 0.3	4.0 ± 0.4	1.8 ± 0.3	n.d.	3.0 ± 0.5	1.3 ± 0.2	n.d.	0.1±0.1	n.d.	0.1 ± 0.1
Lettuce salad	23.0 ± 4.8	1.1 ± 0.2	1.3 ± 0.3	1.5 ± 0.3	1.0 ± 0.1	n.d.	1.0 ± 0.3	0.9 ± 0.1	n.d.	0.1 ± 0.1	n.d.	0.1 ± 0.1
Salad of beetroot	50.3 ± 5.6	0.7 ± 0.2	2.0 ± 0.3	8.0 ± 0.5	8.0 ± 0.6	n.d.	2.0 ± 0.4	0.7 ± 0.1	n.d.	0.1 ± 0.1	n.d.	1.0 ± 0.3
Cauliflower puree	24.7 ± 3.5	0.5 ± 0.1	1.8 ± 0.1	2.1 ± 0.3	2.1 ± 0.4	n.d.	2.3 ± 0.3	1.5 ± 0.2	n.d.	0.1 ± 0.1	n.d.	0.1 ± 0.1
Vegetable puree	67.2 ± 7.7	2.0 ± 0.3	6.0 ± 0.4	4.3 ± 0.5	3.8 ± 0.4	0.5 ± 0.2	3.8 ± 0.5	1.0 ± 0.2	n.d.	0.2 ± 0.1	0.2 ± 0.1	n.d.
Spinach puree	27.8 ± 4.9	1.0 ± 0.3	3.0 ± 0.2	0.5 ± 0.2	0.5 ± 0.1	n.d.	2.4 ± 0.3	1.0 ± 0.2	n.d.	0.1 ± 0.1	n.d.	0.1 ± 0.1
Tomato juice	21.5 ± 4.0	0.2 ± 0.1	1.0 ± 0.2	3.1 ± 0.5	2.7 ± 0.2	0.4 ± 0.2	1.7 ± 0.4	0.5 ± 0.1	n.d.	0.1 ± 0.1	0.2 ± 0.1	0.1 ± 0.1
Peppers	32.3 ± 5.2	0.5 ± 0.1	1.7 ± 0.3	4.2 ± 0.4	4.2 ± 0.3	n.d.	2.0 ± 0.4	1.5 ± 0.2	n.d.	0.1 ± 0.1	n.d.	0.2 ± 0.1

Values are mean and standard deviation.

**Table 3:** Associations between anthrophometric parameters and adipokine levels in the resistant starch (RS) and the Fibre group at the end of the study.

Parameters	RS Group	*p*	Fibre Group	*p*
Body Weight (kg)				
Leptin (ng mL^−1^)	0.458 (0.031, 1.099)	0.039	0.140 (−0.387, 0.686)	0.568
Adiponectin (µg mL^−1^)	0.036 (−1.799, 2.118)	0.865	0.077 (−2.104, 2.824)	0.764
Resistin (ng mL^−1^)	−0.220 (−2.061, 0.653)	0.289	−0.069 (−2.958, 2.188)	0.758
Apelin (µg mL^−1^)	−0.291 (−0.024, 0.005)	0.182	0.139 (−0.014, 0.025)	0.558
Waist Circumference (cm)				
Leptin (ng mL^−1^)	0.315 (−0.189, 0.867)	0.193	0.140 (−0.296, 0.533)	0.559
Adiponectin (µg mL^−1^)	0.064 (−1.690, −2.184)	0.791	−0.131 (−2.389, 1.416)	0.6
Resistin (ng mL^−1^)	−0.059 (−1.508, 1.176)	0.797	−0.104 (−2.447, 1.527)	0.634
Apelin (µg mL^−1^)	−0.225 (−0.021, 0.008)	0.355	0.245 (−0.007, 0.023)	0.295
Body Mass Index (kg m^−2^)				
Leptin (ng mL^−1^)	0.595 (0.227, 1.417)	0.01	0.799 (0.852, 1.629)	<0.001
Adiponectin (µg mL^−1^)	−0.082 (−2.589, 1.774)	0.698	−0.110 (−2.529, 1.036)	0.393
Resistin (ng mL^−1^)	0.108 (−1.124, 1.898)	0.596	0.185 (−0.358, 3.364)	0.108
Apelin (µg mL^−1^)	0.020 (−0.016, 0.017)	0.923	−0.152 (−0.023, 0.005)	0.206

## Data Availability

Data available on request due to restrictions eg privacy or ethical.

## Supplementary Materials

The following are available online at https://www.mdpi.com/2072-6643/13/2/487/s1, Figure S1: Interdependence between anthropometrics parameters (body mass index—BMI, body weight—BW and waist circumference WC) and adipokine levels in RS (RSG) and Fibre group (FG) at the beginning (RSG-0 and FG-0) and end of the study (RSG-12 and FG-12), Figure S2: Interdependence between fibre intake, RS intake and different adipocytokine level (leptin, adiponectin, resistin and apelin), presented as polynomial regression in RS group (Figure S2A,C) and Fibre group (Figure S2B,D).

## Abbreviations

RS	resistant starch
BMI	body mass index
OGT	oral glucose tolerance
DMT2	diabetes mellitus type 2
PA	physical activity
CVs	coefficients of variation
LTPA	Leisure Time Physical Activity
PCA	principal component analysis
SD	standard deviation
CRP	C-reactive protein
TNF	tumor necrosis factor
PA	principal analysis
F	factor-axes
