# Supplementary material for: The Impact of Diet and Fibre Fractions on Plasma Adipocytokine Levels in Prediabetic Adults
Source: Nutrients. 2021 Feb 2;13(2):487. doi: 10.3390/nu13020487 (PMC7913095; doi:10.3390/nu13020487)
Supplement: Supplementary file 1 [file nutrients-13-00487-s001.zip › Supplementary material Figure S1.pdf]

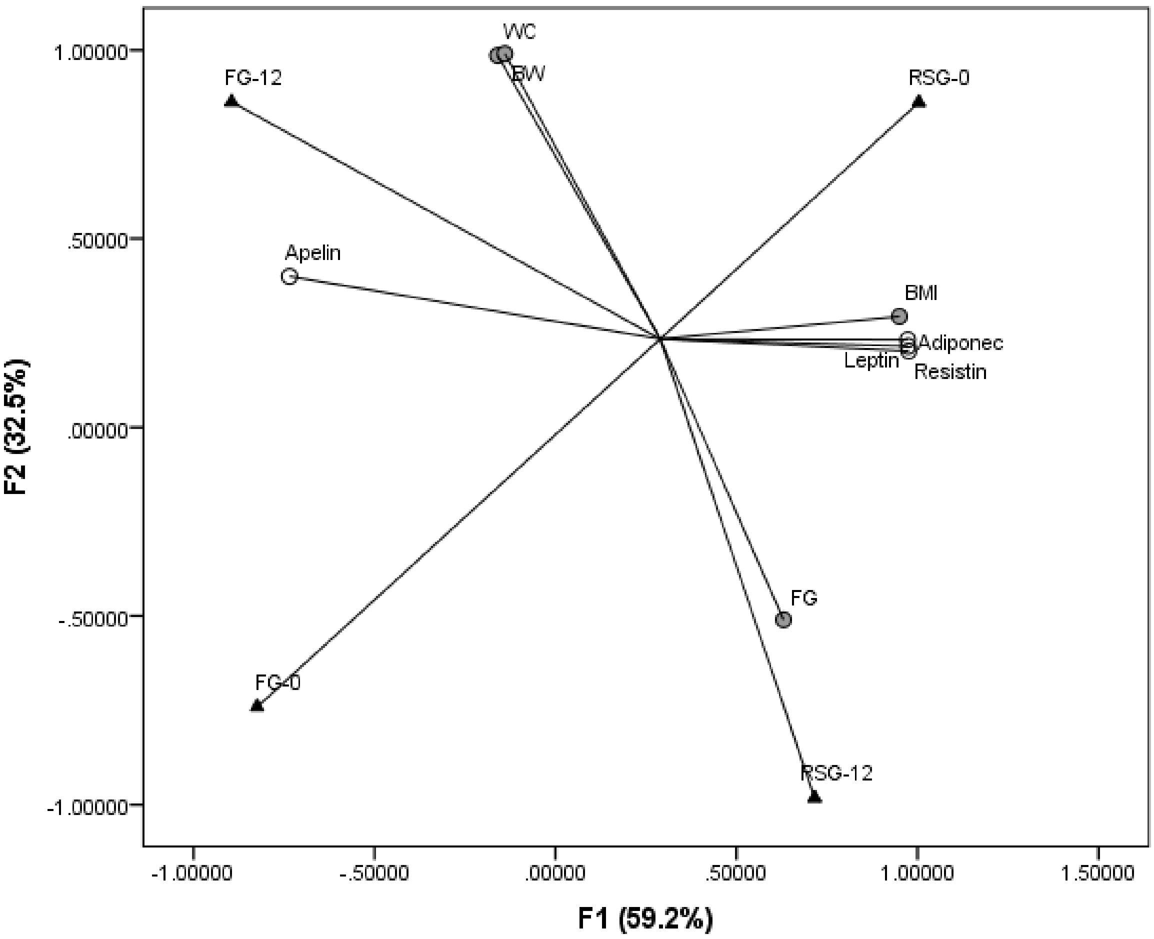

Figure S1**.** Interdependence between anthrophometrics parameters (body mass index - BMI, body weight - BW and waist circumference WC) and adipokine levels in RS (RSG) and Fibre group (FG) at the beginning (RSG-0 and FG-0) and end of the study (RSG-12 and FG-12)
