# Supplementary material for: The Impact of Diet and Fibre Fractions on Plasma Adipocytokine Levels in Prediabetic Adults
Source: Nutrients. 2021 Feb 2;13(2):487. doi: 10.3390/nu13020487 (PMC7913095; doi:10.3390/nu13020487)
Supplement: Supplementary file 1 [file nutrients-13-00487-s001.zip › Supplementary material Figure S2.pdf]

A

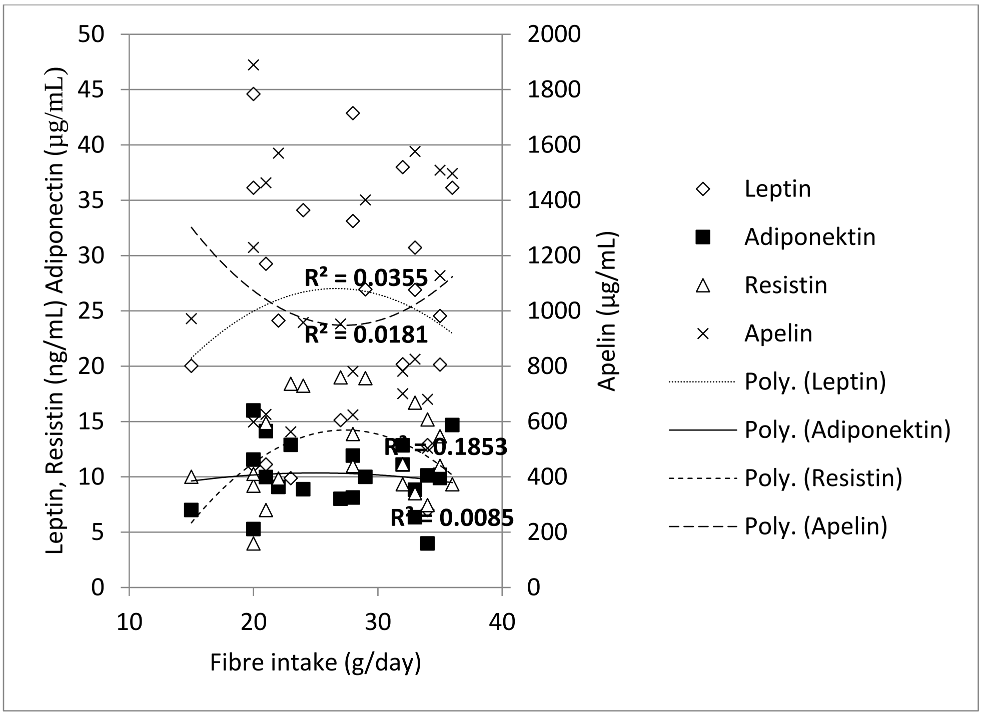
Leptin, Resistin (ng/mL) Adiponectin (µg/mL)
Apelin (µg/mL)
Fibre intake (g/day)
R² = 0.0355
R² = 0.0181
R² = 0.1853
R² = 0.0085
Leptin
Adiponektin
Resistin
Apelin
Poly. (Leptin)
Poly. (Adiponektin)
Poly. (Resistin)
Poly. (Apelin)

B

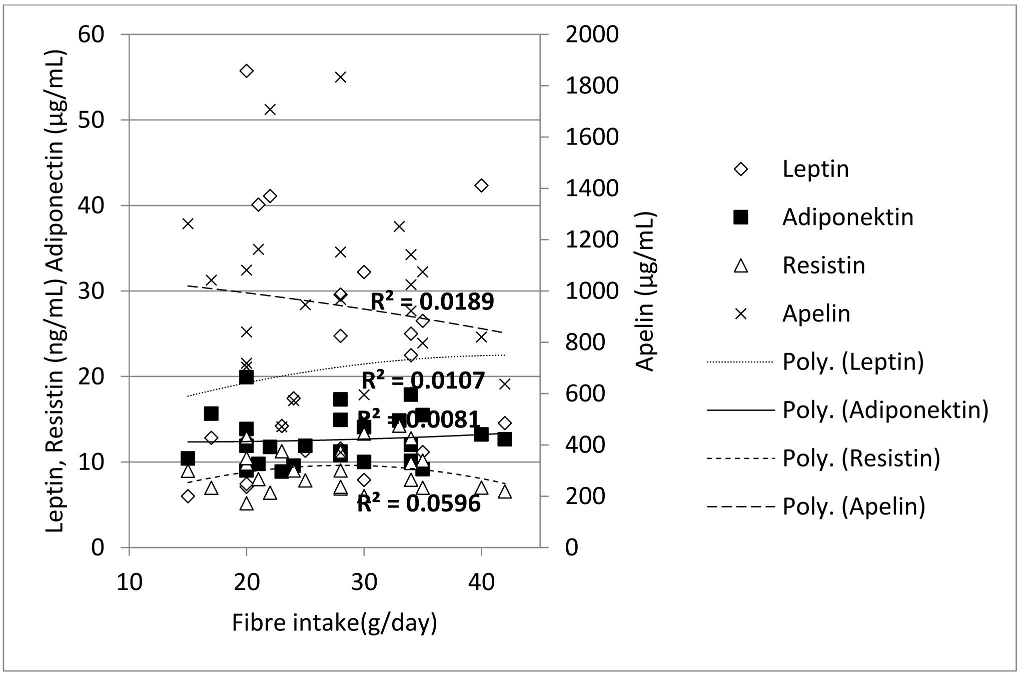
Leptin, Resistin (ng/mL) Adiponectin (µg/mL)
Apelin (µg/mL)
Fibre intake(g/day)
R² = 0.0189
R² = 0.0107
R² = 0.0081
R² = 0.0596
Leptin
Adiponektin
Resistin
Apelin
Poly. (Leptin)
Poly. (Adiponektin)
Poly. (Resistin)
Poly. (Apelin)

C

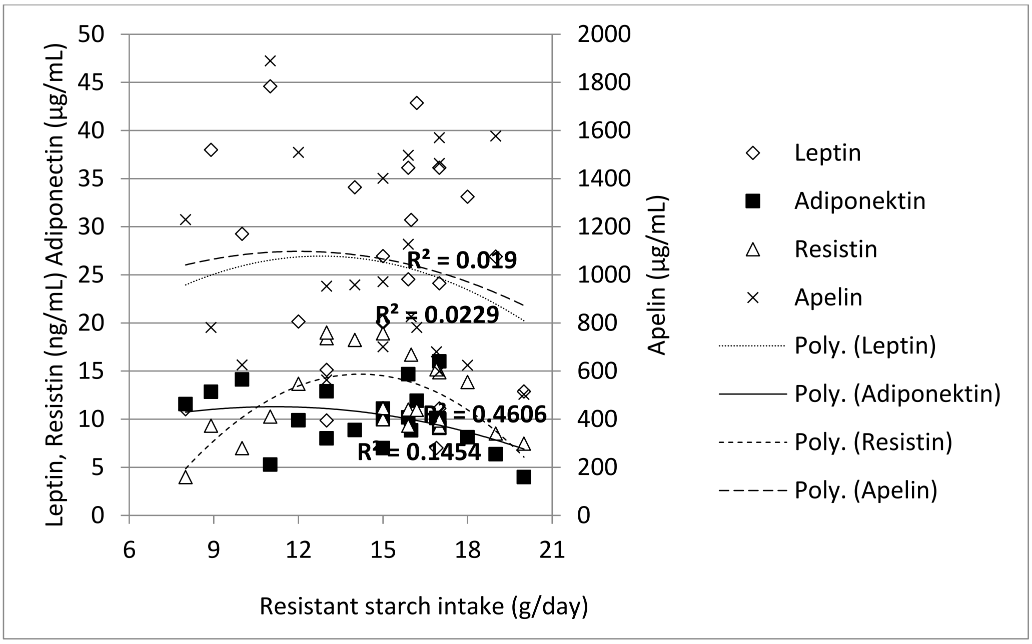

D

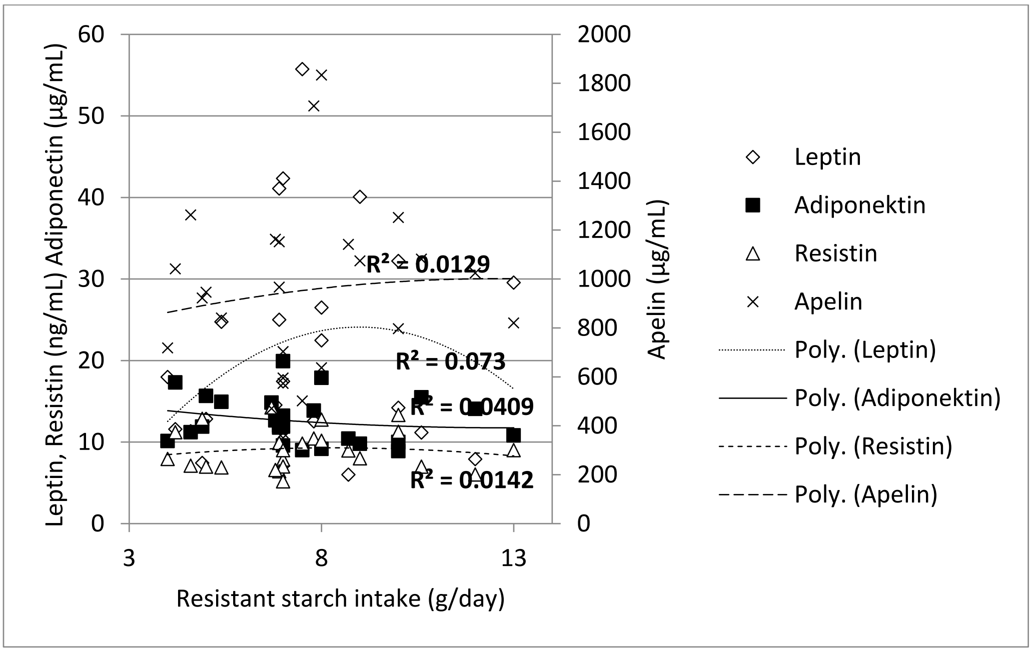

Figure S2 (A, B, C, D). Interdependence between fibre intake, resistant starch intake and different adipocytokine level (leptin, adiponectin, resistin and apelin), presented as polynomial regression in RS group (A, C) and Fibre group (B, D).
